# Implant selection in natural and stable direct-to-implant reconstruction with ten steps at nipple-sparing mastectomy

**DOI:** 10.1097/MD.0000000000033758

**Published:** 2023-05-12

**Authors:** Mehmet Sağir, Erdem Güven, Seda Eröz, Cihan Uras

**Affiliations:** a Specialist in Department of Plastic Surgery, Private Acibadem Maslak Hospital, Sariyer, İstanbul, Turkey; b Specialist in Department of Radiation Oncolog, Private Acibadem Maslak Hospital, Sariyer, İstanbul, Turkey; c Specialist in Department of General Surgery, Private Acibadem Maslak Hospital, Sariyer, İstanbul, Turkey.

**Keywords:** breast cancer, breast reconstruction, direct-to-implant reconstruction, immidiate breast reconstruction, niple-sparing mastectomy

## Abstract

Direct-to-implant reconstruction is one of the breast repair techniques after mastectomy. Implant selection is critical in the short- and long-term success of direct-to-implant reconstruction after nipple-sparing mastectomy. In this study we developed a 10-step algorithm that we use before and during surgery. We aimed to obtain natural and stable breast reconstruction with this algorithm. In addition, we also aimed to evaluate which implants were selected using this algorithm and their short- and long-term outcomes. This retrospective study included 218 patients aged 27 to 60 years who underwent mastectomy and direct-to-implant reconstruction between November 2018 and December 2021. The patients were assigned into 4 groups according to amount of breast tissue removed. We developed a 10-step algorithm and these included: breast base, amount of breast tissue removed, evaluation of mastectomy skin flap, breast projection, ptosis, unilateral/bilateral reconstruction, chest wall deformity, patient’s request, comorbid conditions and stabilization and arrangement of novel sulcus. The evaluation was made when the patient’s photographs were taken at least 1 year after the surgery. The highest number of patients was recorded in group 3; in addition, mean age was also highest in group 3. The lowest number of patients was recorded in group 4. The body mass index showed a progressive increase from group 1 to group 4. Medium height moderate profile prosthesis was used in 81.7% while medium height moderate plus profile prosthesis was used in 18.3% of breasts included. We used larger prosthesis up to 58.1% when compared to the tissue removed in group 1 while we used smaller prosthesis by 25.6% in group 4. In the anterior view, the medial and lateral arch of the lower pole of the breast was obtained in all patients. Obvious asymmetry developed in 4 patients. In lateral and oblique views, upper and lower pole natural breast images were obtained in all patients, except for 5 patients. There was no sulcus inferior displacement in any patient. Implant extrusion did not occur in any patient. This algorithm is an easy to use and effective method to obtain a stable and natural breast image in the long-term.

## 1. Introduction

Breast cancer is the most common non-cutaneous cancer in women.^[1]^ The breast reconstruction has been increasingly performed following cancer surgery. Studies have shown that breast reconstruction has positive contributions to the psychology of women.^[2]^

Many techniques have been described for breast reconstruction following mastectomy. Compared to autologous tissue reconstruction, implant-based reconstruction is less invasive and less morbid but more expensive.^[3–5]^ The priority in breast reconstruction is to achieve satisfactory aesthetic result and low complication.^[6]^ Using a breast implant for reconstruction can restore the natural feel, size and shape of the breast.^[1]^

Reconstruction after nipple-sparing mastectomy (NSM) requires the correct formation of the breast shape and nipple position.^[7]^ Thus, implant selection is critical in the short- and long-term success of NSM.

In NSM, it is tried not to leave behind as much breast tissue as possible. The skin flaps can be extremely thin since the skin flap is small in radical mastectomy.^[8]^ Given the burden on the skin and longer skin flap, the risk for necrosis is higher in NSM.^[8]^

In the literature, there is no algorithm for implant selection in patients undergoing NSM. In order to fill this gap, we developed a 10-step algorithm that we use before and during surgery. We aimed to obtain natural and stable breast reconstruction with this algorithm. In addition, we also aimed to evaluate which implants were selected using this algorithm and their short- and long-term outcomes.

## 2. Materials and methods

This retrospective study included 218 patients aged 27 to 60 years who underwent mastectomy and immediate implant-based breast reconstruction between November 2018 and December 2021. Patients who received radiotherapy and who used skin reduction techniques were excluded from the study. If the tissue removed from the 2 breasts in bilateral reconstruction fit different groups, these patients were also excluded from the study. Table 1 presents demographic data of the patients. Of the patients included, 196 had given birth and breastfed their infants.

**Table 1:** Demographic characteristics of patients.

Groups	Number of patients	Age (average)	BMI	Smoking		Cancer side			Comorbidity (number of patients)
				Active smoking	Ex smoking	Right	Left	Prophylactic	
0–200 cc	40	44.7	21.6	8	9	25	15	2	Cardiovascular disease (3)thyroiditis (4)
201–350 cc	66	41	22.9	8	15	22	36	8	Thyroiditis (3)hypertension (2)collegene tissue disease (1)
351–650 cc	91	47.1	26.1	21	17	48	36	8	Diabetes (6)hypertension (4)collegene tissue disease (2)
>650 cc	21	44.1	31.1	4	5	12	10	-	Diabetes (2)hypertension (1)thyroiditis (2)
Total	218	44.2	25.4	41	46	106	98	18	30
Average				18.8%	21.1%	48.6%	44.9%	8.2%	13.7%

BMI = body mass index.

The patients were assigned into 4 groups according to amount of breast tissue removed:

Group 1: 0 to 200 cubic centimeters (cc)

Group 2: 201 to 350 cc

Group 3: 351 to 650 cc

Group 4: 651 cc or greater

### 2.1. Assessment

**Figure 1.**
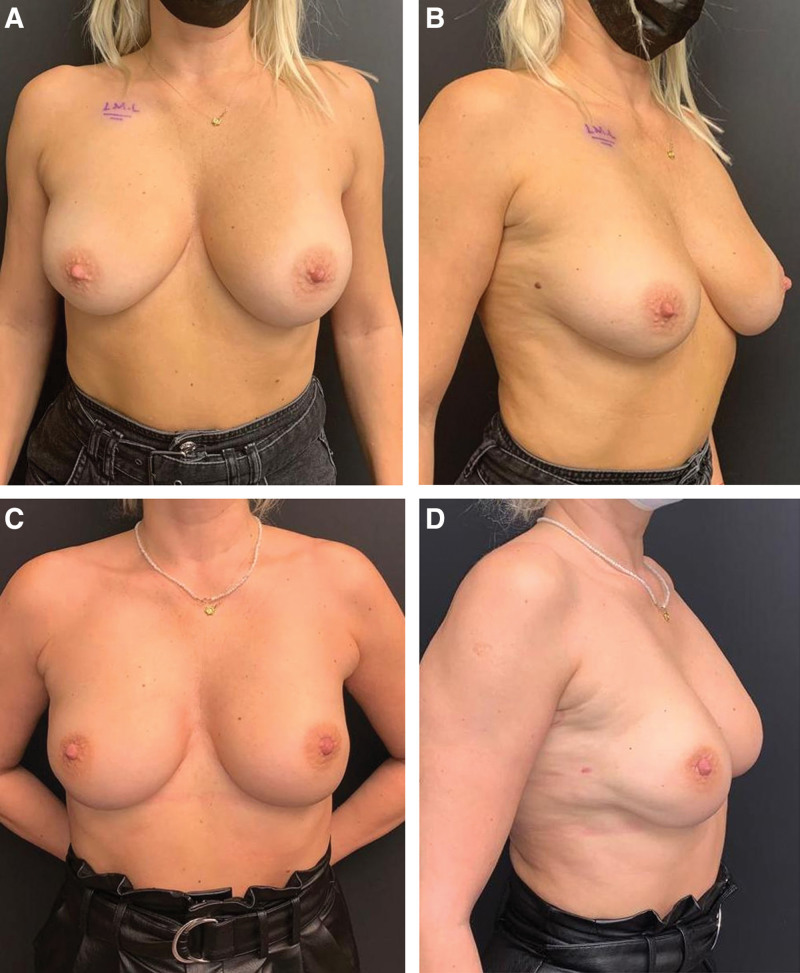
50-year-old multiparous with right breast cancer patient underwent bilateral NSM + direct-to-implant reconstruction. She has high-stiffness skin structure and asymmetric breast pattern and is in group 3. Reconstruction was performed with an IMF insicion. 490 cc of tissue from the right breast and 580 cc of tissue from the left breast were removed. Reconstruction with 480 cc medium height moderate profile anatomical implant. (A) Preoperative anterior view. (B) Preoperative oblique views. (C) Postoperative 14th month anterior view. The same model implant was placed on both breasts and postoperative good symmetry was achieved. There was a certain amount of shrinkage in the left breast. (D) Postoperative 14th month oblique view. IMF = Inframammary fold, NSM = Nipple-sparing mastectomy.

**Figure 2.**
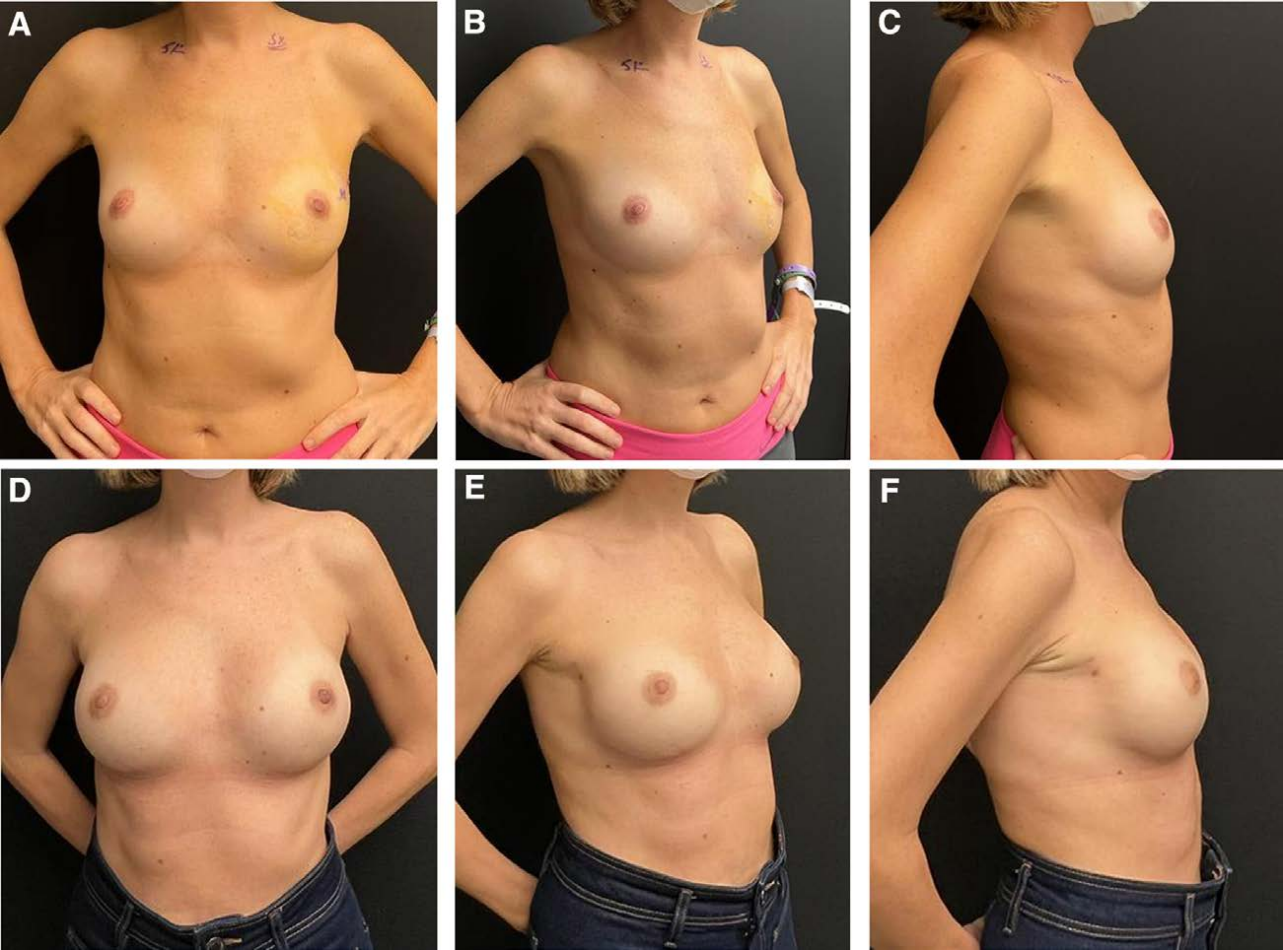
41-year-old multiparous with left breast cancer patient underwent NSM + direct-to-implant reconstruction. She has high-stiffness skin structure and is in the group 1. Reconstruction was performed with an IMF incision. 186 cc of tissue was removed from each breast. Medium height modorate profile 280 cc implant was used for both breasts. (A) Preoperative anterior view. (B) Preoperative oblique view. (C) Preoperative lateral view. (D) Postoperative 12th month anterior view. In the medial and lateral quadrants, it is seen that the natural breast curve of the breast is formed and good symmetry was achieved. (E) Postoperative 12th month oblique view. IMF incision scar is shown. (F) Postoperative 12th month lateral view. It was shown that the lower and upper quadrants of the breast were formed in the Figure 2E and Figure 2F. Also, the location, stabilty and symmetry of the inframmaryal sulcus are shown. IMF = Inframammary fold, NSM = Nipple-sparing mastectomy.

**Figure 3.**
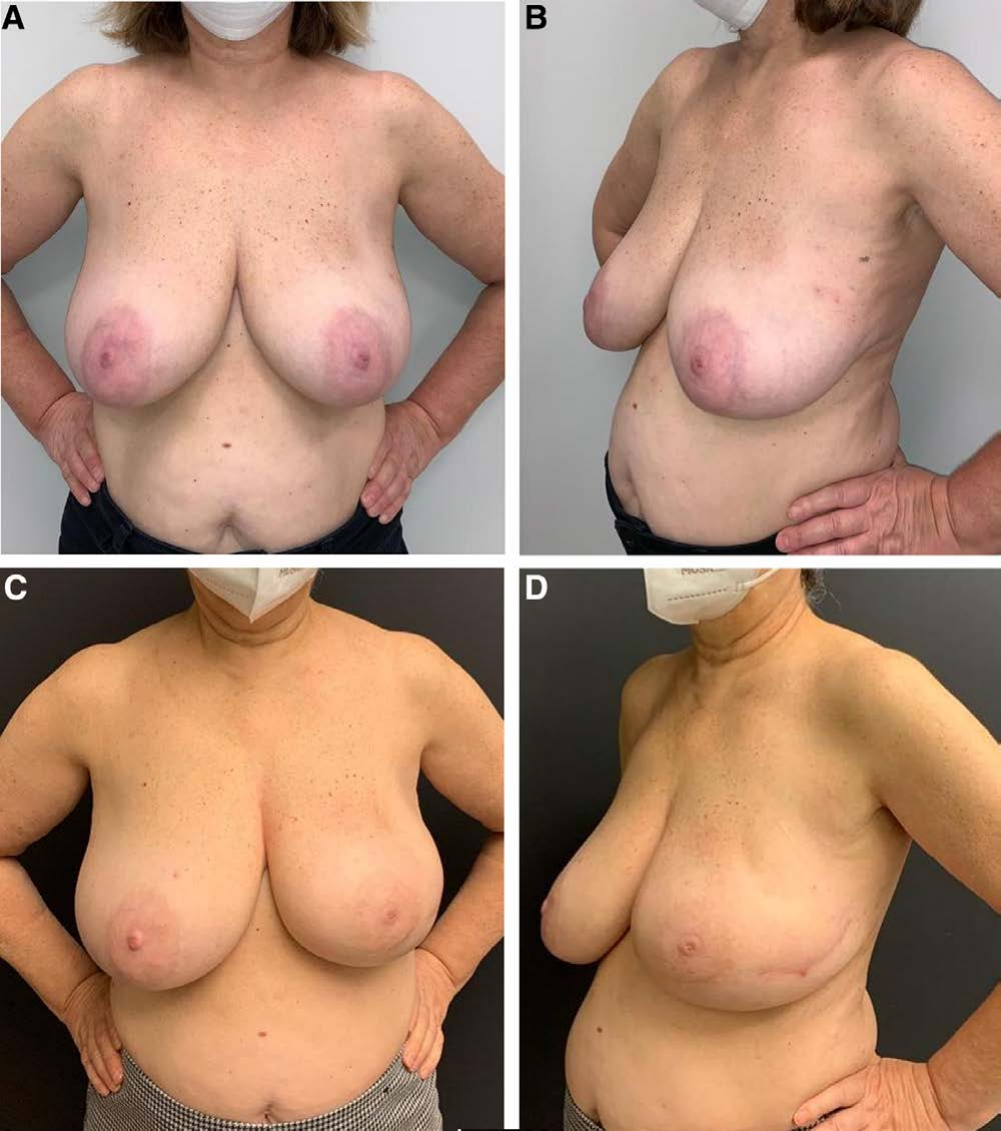
60 years old multiparous with left breast cancer patient underwent NSM + direct-to-implant reconstruction. She has low-stiffness skin structure and is in the group 3. Reconstruction was performed with an lateral curved incision. 640 cc of tissue was removed from the left breast. Breast reconstruction with medium height modorate profile 530 cc implant was done. (A) Preoperative anterior view. (B) Preoperative oblique views. (C) In the postoperative 26th month image, it was shown that medial and lateral quadrant of the breast was created. A certain amount of reduction was seen in the left breast. Symmetry is not fully achieved. (D) In the postoperative 26th month oblique view, it was shown that the upper pole and lower pole were formed. The location of the lateral incision is also shown in this image. NSM = Nipple-sparing mastectomy.

The evaluation was made when the patient’s photographs were taken at least 1 year after the surgery by the operating nurse and the surgeon’s assistant. Anterior, oblique and lateral views were evaluated. In anterior views, it was checked whether a natural breast curve was formed in the medial and lateral of the lower pole of the breast and whether the prosthesis filled the base of the breast (Figs. 1C, 2D, 3C). In the oblique and lateral views, the formation of the upper and lower poles of the breast and displacement of the sulcus were evaluated (Figs. 1D, 2E, 2F, and 3D). Also, postoperative obvious asymmetry was evaluated. The radiological image was performed at the unnatural breast appearance because of the possibility of prosthesis rotation or capsular contracture.

### 2.2. Surgical procedure

Lateral curved and inframammary fold incisions were used in all patients (Figs. 3D, 2E). Dual and prepectoral plans were used as the implant site. The biological mesh was used to support the prosthesis segment uncovered with the pectoralis muscle. In the prepectoral reconstruction, the prosthesis was wrapped in biological mesh and sutured to the chest wall. An anatomic, textured implant (Mentor Medical System B.V. Zernikedreef 2 2333 CL Leiden) was used. Two types of implants were used including medium height moderate (Mentor Contour Profile Gel 321; Cohosive III Gel Breast Implant, Medium Height, Moderate Profile) and moderate plus profile (Mentor Contour Profile Gel 322; Cohosive III Gel Breast Implant, Medium Height, Moderate Plus Profile).

When selecting prosthesis to be used in the patient, the following parameters were assessed respectively:

1. Breast base: The first parameter to be considered in the selection of the prosthesis is the breast base. In all patients, the breast base was measured before surgery. During surgery, a pocket was prepared based on the breast base and final adjustments were made using a sizer.
2. Amount of breast tissue removed: Volume was measured by placing removed tissue into a water-filled container. In bilateral reconstruction, breasts were measured separately.
3. Evaluation of mastectomy skin flap: In the preoperative and intraoperative evaluation, the breast skin structure was divided into two as high-stiffness and low-stiffness.^[9]^ In the preoperation, the patient’s birth or breastfeeding and weight-loss history were questioned. Again, skin flap was evaluated after mastectomy during the operation and the decision was made exactly as a result of this evaluation. We inserted our hand into the mastectomy pouch from the incision and pushed the skin outward with maximum force. It was defined as low-stiffness for a mastectomy flap that has low skin resistance and can be expanded easily. High-stifness was defined for the flap with high skin resistance and limited skin expansion. High-stiffness mastectomy skin flap structure is shown in Figure 1A–D and Figure 2A–F. Low-stiffness skin flaps is shown in Figure 3A–D.
4. Breast projection: The implant selection was made based on breast projection upon breast base and distance between nipple and sulcus according to preoperative measurements.
5. Ptosis and asymmetry: In the presence of ptosis, we do not recommend breast reduction procedures in group 1. In group 2, inverted-T scar technique was recommended to patients with grade 2 or 3 ptosis based on body form and jugulum-nipple distance. In groups 3 and 4, inverted-T scar technique was recommended in the presence of grade 2 and 3 ptosis. The patients underwent inverted-T scar technique were excluded. In patients declining skin reduction, the biological mesh size was adjusted according to the grade of ptosis (Figs 1A–D, 3A–D). If skin reduction techniques were used in the asymmetric breast, these patients were excluded from the study. In groups 1 and 2 in the asymmetric breast, which was not used skin reduction techniques, implants were selected according to the large breast and the same model and size implant was used for the small breast. In groups 3 and 4, we selected implants according to the small breast and used the same model and size implant for the other breast (Fig. 1A–D).
6. In asymmetry, if the breasts are from a separate group, these patients were excluded from the study because of disruption of distribution of patients in the groups.
7. Unilateral/bilateral reconstruction: In unilateral reconstruction, the disease-free breast was taken as reference. The grade of ptosis in contralateral breast dictates shape of the breast undergoing reconstruction. In bilateral reconstruction, larger or smaller prosthesis can be used in both breasts according to the algorithm (Figs. 1A–D, 2A–F).
8. Chest wall deformity: If it causes obvious asymmetry in the breast, it was excluded from this study. If the ribs in the projection of the unilateral breast are depressed compared to the other side, the moderate plus implant was preferred, if more protruding, the moderate implant was preferred. The moderate plus implant was preferred if the ribs in the bilateral breast projection were depressed, and the moderate implant was preferred if they were more protruding.
9. Comorbid condition: In the presence of comorbid conditions (including diabetes mellitus, collagen tissue disorders, cardiovascular disorders etc) that may interact with wound healing and circulation of skin flap, we selected prosthesis which will not lead tension on skin flaps.
10. Stabilization and arrangement of novel sulcus: Sulcus asymmetry, if any, was determined between the breasts. The sulcus was placed at an equal level in both breasts. In each patient, the sulcus was stabilized with a polydioxanone suture suture. In addition, we supported the stabilization of the sulcus by stitching the lower edge of the biological mesh to the sulcus. The location and symmetry of the sulcus after reconstruction are shown in Figure 2D–F.
11. Patient’s request: The patient’s requests were assessed in the context of the algorithm.

Two drains were placed to each breast. Antibiotic therapy and analgesics were given until drain removal. The patients did not wear a bra for the first 2 weeks.

Ethics approval was obtained by the Acibadem University Ethics Committee (ATADEK) (2023-06/208). Informed consent was obtained from all patients.

## 3. Results

Mean follow-up was 23 months. A total of 33 (15.1%) of the patients were prepectoral and 185 (84,8 %) were dual plan. The highest number of patients was recorded in group 3; in addition, mean age was also highest in group 3. The lowest number of patients was recorded in group 4. The body mass index (BMI) showed a progressive increase from group 1 to group 4.

Table 2 presents prosthesis used in the patients and distribution of unilateral and bilateral reconstructions. Unilateral reconstruction was performed in 49.5% of the patients. Medium height moderate profile prosthesis was used in 81.7% while medium height moderate plus prosthesis was used in 18.3% of breasts included. On average, prosthesis larger than tissue removed in the mastectomy was used in group 1 and 2 while prosthesis smaller than tissue removed in the mastectomy was used in group 3 and 4. We used larger prosthesis up to 58.1% when compared to the tissue removed in group 1 while we used smaller prosthesis by 25.6% in group 4.

**Table 2:** Distribution of the implant used according to the groups.

Groups	Unilateral reconstruction patients (n)	Bilateral reconstruction patients (n)	Medium height moderate profile implant (n)	Medium height moderate plus profile implant (n)	Unilateral reconstruction (average)		Bilateral reconstruction (average)		Total (average)	
					Removed tissue	The prosthesis placed	Removed tissue	The prosthesis placed	Removed tissue	The prosthesis placed
Group 1 0–200	22	18	49	9	167.3	217.2	151.6	268.8	157.6	249.3
					Increased 29.8%		Increased 77.8%		Increased 58.1%	
Group 2 201–350	38	28	77	17	275.4	281.5	273.3	316.7	274.2	302.5
					Increased 2.2%		Increased 15.8%		Increased 10.3%	
Group 3 351–650	33	58	123	26	480.1	443.7	477.2	444.1	476.5	444
					Decreased 7.5%		Decreased 6.9%		Decreased 6.8%	
Group 4 >650 cc	15	6	19	8	769.3	560	748	570	760.2	565.2
					Decreased 27.2%		Decreased 23.7%		Decreased 25.6%	
Total (n) (%)	108	110	268	60						
	% 49.5	% 51.5	% 81.7	% 18.3						

Of 328 breasts included, 256 (78%) showed low-stiffness skin. The lowest number of breasts with low-stiffness skin was recorded in group 1. When the groups were evaluated within themselves, high-stiffness was found in group 1 the most.

In the anterior view, the medial and lateral arch of the lower pole of the breast was obtained in all patients, except for 5 patients. Obvious asymmetry developed in 2 patients in group 1 and 2 patients in group 4, which was performed unilaterally. Ninety-three cc of tissue was removed from one of the patients in group 1 and 115 cc of tissue from the other. In group 4, 720 cc of tissue was removed from 1 patient and 680 cc of tissue from the other. In lateral and oblique views, upper and lower pole natural breast images were obtained in all patients, except for 5 patients. Magnetic resonance imaging results in 2 patients showed that the implant returned, and capsule contracture developed in 3 patients. These patients were taken to revision surgery. There was no inferior displacement of sulcus in any patient.

The circulation disorder or necrosis was separately assessed form skin flap and nipple-areola complex (NAC). The necrosis was treated with intervention in 10 breasts, resulting no implant loss. In skin flaps, primary repair was performed in 8 while local flap was used for repair in 2. Of the necrosis at NAC, 2 involved whole NAC while remaining involved <10% of NAC. Medium height moderate profile prosthesis was used in all patients with necrosis at NAC. In the patient with complete NAC necrosis, the necrosis was removed, and defect was closed primarily. In cases with partial NAC necrosis, primary repair was performed due to small defect. No implant change was performed in these patients.

Necrosis and implant loss were observed in 3 breasts. Bilateral necrosis involving one-fourth of total breast areas and subsequent implant loss was developed in a patient (group 3) with tuberous breast pattern who had history of smoking and hypertension and underwent bilateral reconstruction using medium height moderate plus implant. Again, necrosis involving 9% of total breast area and subsequent implant loss was developed in a patient with BMI of 35.1 and history of smoking who underwent unilateral reconstruction. A medium height moderate profile prosthesis was used in this patient. Early complications are given in Table 3.

**Table 3:** Early complications.

Early complications (per breast)			
	Number	Ratio %	
Full thickness NAC necrosis	5	1.5 %	Excision of necrotic tissue and primary repair were performed.
Full tickness mastectomy flap necrosis	10	3.04 %	Necrosis was at the incision line in 5 breasts and at the skin flap in 3 breasts. Excision of necrotic tissue and primary repair were performed. The necrosis of the skin flap in 2 breasts was repaired with a local flap.
Reconstruction failure	3	0.9 %	At the end of the adjuvant treatments, reconstruction was done with the latissimus dorsi flap + the implant.
Infection	8	2.4 %	The patients were treated with IV antibiotics.
Seroma	5	1.5 %	Seroma was aspirated with the help of USG.
Hematoma	3	0.9 %	It was evacuated in operating room conditions.

IV = intraveneous, NAC = niple aerola complex.

Implant extrusion did not occur in any patient. Fat injection was performed in depressed areas in 6 patients.

## 4. Discussion

Today, silicone implants are more popular than saline implants due to more natural appearance and breast feeling.^[10]^ The textured implant has tendency to maintain its location at long-term by attaching tissue; however, this is generally not valid in smooth implants.^[11]^ Since the goal was to achieve natural breast reconstruction in our study, we used anatomic, textured, silicone implants in all patients.

In breast reconstruction, the breast base width and amount of tissue removed are primary factors for implant selection.^[10]^ In the selection of implants that do not fill the base of the breast, an unnatural breast image can occur, and the nipple position can rotate laterally. Although the amount of tissue removed from the breast and the base of the breast consists of the basis of prosthesis selection, we think that there are other factors besides these. We argue that the 10-step algorithm we present is the steps to be followed for permanent and natural breast reconstruction in patients who skin reduction techniques are not applied, and who do not receive radiotherapy.

The breast base width can be measured using a ruler or 3-dimensional volumetric computer software before surgery.^[12]^ In our study, no imaging modality was used to measure breast base width. We have different sizes of implants and sizers available in the operating room. We advocate that final decision in implant selection should be made after establishing nipple location and natural breast appearance during surgery.

The Cooper ligament is the structure exposed to highest load of soft tissue within breast; followed by skin.^[13]^ On the other hand, inferior pole and lateral fascia are thicker.^[14]^ This is the body’s response to gravity when supine and standing position.^[9]^ Since most of these ligaments and fascia structures are removed in NSM, we advocate that some of the prosthetic weight be transferred to the chest wall with the help of mesh in the early period of direct-to-implant reconstruction.

The implant applies continuous pressure on the inferior pole of the breast through gravity and dynamic daily activity.^[9]^ In a previous study, it was emphasized that, in mastopexy, the large implant with high projection lead parenchymal atrophy and tissue thinning in breast.^[15]^ In breast reconstruction, long-term pressure of the prosthesis on the lower pole of the mastectomy flap with the effect of gravity may cause thinning of the lower pole flap, prosthesis exposure and implant loss. Metere et al^[16]^ observed prosthesis extrusion in 3.8 % of patients in their long-term follow-up. We took 2 steps to prevent this complication. These; using biological mesh and moderate/moderate plus profile prosthesis. Mesh forms a layer on the lower pole and increases the distance between the epidermis and the prosthesis in the lower pole. The fact that we did not see implant extrusion in any of our patients shows the effectiveness of our steps.

Many methods have been used to assess breast volume before surgery, including mammography, magnetic resonance imaging, Archimedes principle and anthropometric measurements.^[17]^ While some of them are not cost effective, some of them are measurements that contain some calculation formulas.^[17]^ These methods can be convenient for surgeons in breast reduction, breast augmentation and mastopexy. However, in mastectomy, the existing breast tissue is removed. It is a simple method to determine the volume of the breast tissue removed with a water container in surgery.

Subcutaneous adipose tissue shows undulation within breast and the distance between dermis and breast tissue ranges from 8 mm to 14 mm.^[8]^ This means that the dissection brings some areas closer to the skin, and as a result, depressed areas may occur in the skin flap. Fat injection was recommended in the displaced areas in our patients.

Subcutaneous adipose tissue is thicker in elder and patients with high BMI.^[18]^ In a previous study, the mean skin thickness was found as 3.1 mm ranging from 1.5 mm and 5.0 mm.^[8]^ In our study, we did not perform dermis thickness and subcutaneous adipose tissue quantitatively; rather, we used the skin assessment described by Vegas and Yerro.^[9]^ They described the category of low-stiffness skin as the breast group with poor tissue support due to birth and breastfeeding.^[9]^ On the other hand, our clinical experience was that some patients who gave birth had high-stiffness mastectomy skin flap structure (Fig. 1A–D). Authors suggested that there may be ptosis-related complications due to poor tissue support over time when large implant with high projection is used.^[9]^ For this group, they recommended the use of a moderate sized/projected prosthesis in patients with mastopexy.^[9]^ The fact that mean age was 44.2 years in our patients and 89% had given birth explains low-stiffness skin flap in 78% of our patients. Our use of medium height moderate profile implants with a rate of 81.7% in our study was consistent with this information in the literature. The high-stiffness category included patients without history of birth or those with skin structure seen in tuberous breast deformity.^[9]^ Authors suggested that augmentation in breast will provide better long-term outcomes.^[9]^ In our study, the patients in group 1 had the most high-stiffness structure and showed that a larger volume of implants could be used safely with an average of 58.1 % of the removed tissue (Fig. 2A–F). On the other hand, in patients with low or high-stiffness skin, in whom a smaller prosthesis is used than the tissue removed, the skin shrinks a little in the long-term (Figs. 1A–D, 3A–D). The natural breast image is not distorted and less load is placed on the lower pole. In the low-stiffness skin structure, especially in the lower pole, the skin may become thinner over time and the risk of extrusion of the prosthesis should be kept in mind.

Skin flap necrosis is the most serious complication of NSM.^[19]^ In the literature, the necrosis rate ranges from 5% to 30% in skin flaps.^[20]^ In our study, full thickness mastectomy skin flap necrosis was observed in 5.4% of breasts (Table 3). Radu et al^[18]^ proposed that mastectomy skin flap thickness alone is responsible from necrosis in flap and NAC. In a study by Robertson et al^[20]^, it was concluded that smoking, diabetes mellitus, obesity, radiotherapy, previous surgical scar, severe medical comorbidity were associated with partial or full thickness flap necrosis. In another study, smoking, younger patients, and incision type (periaerolar or superior circumaerolar) were found to be high-risk group.^[21]^ Lemaine et al^[22]^ classified the circulatory problem in the mastectomy flap according to depth and surface area. In this classification, A1 was the best and D4 was the worst.^[22]^ Although the aim in reconstruction is to keep all patients in class A1, this is not always possible. Given that NSM has been performed since 2001,^[19]^ we think that the necrosis rate in the literature will be stabilized and decreased over time by accumulating experience with NSM and selecting the appropriate prosthesis according to the patient.

In the study of Frey et al^[23]^, the rate of patients who had an infection and treated with intraveneous antibiotics was 1.7%, and the rate of implant failure was 3.9%. In the study of Choi et al^[24]^, they found these rates to be 2.5% and 5.6%, respectively. In our study, this rate was 2.4% and 0.9%, respectively. We think that our low implant loss is an indicator of the efficiency of our algorithm. It should also be considered that we excluded patients who received radiotherapy from the study.

In our study, the sulcus was stabilized with a polydioxanone suture suture. Moreover, suturing the lower border of the biological mesh to the sulcus provided double-supported protection in the sulcus and created a more stable sulcus in the long-term (Fig. 2D–F). On the other hand, it is important to determine the sulcus asymmetry of both breasts and to plan an intervention for this before the operation.

We advocate the use of skin reduction techniques for the patient population in Group 4. The possible risk of NAC necrosis in the inverted-T scar technique discourages some of the patients from this technique. The reason why the least number of patients were in group 4 was that we recommended skin reduction procedures to the patients in this group.

We cannot predict how radiotherapy will affect the breast. In the long-term, we cannot distinguish whether this deformity is caused by the algorithm or the effect of radiotherapy. Therefore, these patients were excluded from the study. Again, the algorithm to be followed in breast reconstruction with skin reduction is the subject of a different study.

Our study has some limitations. This is a retrospective study. Which implant will be chosen is determined exactly in the surgery. Therefore, implants of different sizes should be available in the operating room. Evaluation of skin flap quality (low-stiffness, high-stifness) is subjective.

## 5. Conclusion

Results showed that the implant selected according to this algorithm in certain patient groups provides permanent and natural breast reconstruction. It was concluded that medium height moderate profile implant should be used in the majority of this group of patients while medium height moderate plus profile implant should be used in selected patients. Again, our study showed that the breast can be enlarged according to the algorithm and it can be reduced to some extent by placing a smaller prosthesis without any excision from the skin.

## Author contributions

**Formal analysis:** Mehmet Sağir, Seda Eröz.

**Resources:** Mehmet Sağir.

**Supervision:** Mehmet Sağir, Erdem Güven, Cihan Uras.

**Validation:** Cihan Uras.

**Writing – original draft:** Mehmet Sağir.
